# Characteristic and resource potential of water soluble lithium in lithium-rich salt lake sediments from Qaidam Basin, China

**DOI:** 10.1371/journal.pone.0336483

**Published:** 2025-11-07

**Authors:** Zhe Ma, Xiuzhen Ma, Dan Ma, Yubin Li, Xin Liu, Guojing Zhu, Qi Wei, Yunqi Ma, Syed Hussain Asim, Lu Cong

**Affiliations:** 1 Key Laboratory of Green and High-end Utilization of Salt Lake Resources, Qinghai Institute of Salt Lakes, Chinese Academy of Sciences, Xining, China; 2 Xining Center of Natural Resources Comprehensive Survey, China Geological Survey, Xining, China; 3 Construction Materials Testing Laboratory, Public Works Department, Gilgit, Pakistan; 4 Chengdu Academy of Educational Sciences (Chengdu Research Office of Basic Education), Chengdu, China; Institute of Urban Environment Chinese Academy of Sciences, CHINA

## Abstract

This study investigates geochemical characteristics and resource potential of water-soluble lithium in lithium-rich salt lake sediments from the Qaidam Basin, focusing on the East Taijinar Salt Lake and Bieletan area. Through X-ray diffraction (XRD), elemental analysis (ICP-OES), and laser particle size analysis (LPSA), sediment samples were analyzed to assess mineral composition, lithium distribution and its occurrence forms. Results reveal distinct vertical zonation: lithium, boron, and potassium peak in clay-rich layers, contrasting with lower concentrations in halite-dominated layers. Regional patterns indicate that lithium enrichment in sediments aligns closely with brine, centered around Yiliping and East/West Taijinar Salt Lakes. Water-soluble lithium primarily originates from weak adsorption on clay minerals, with secondary contributions from intercrystalline brine, halite fluid inclusions, and gypsum dissolution. The clay layers exhibit lithium concentrations exceeding industrial grade and favorable Mg/Li ratios, comparable to brine mining standards. Co-enrichment of boron (415 ppm) and potassium highlights multi-resource potential. These findings highlight sediments as lithium reservoirs, which can serve as a sustainable potential supplement during brine depletion and enhance resource resilience in the Qaidam Basin. This study provides critical insights into lithium migration, enrichment mechanisms, and strategic resource management in evaporitic systems.

## 1. Introduction

Lithium (Li), the lightest metallic element and a critical enabler of the modern energy transition, is indispensable across numerous industries. Its high electrochemical potential and low density render it essential for rechargeable lithium-ion batteries (LIBs), which are dominant in electric vehicles (EVs), portable electronics, and grid-scale energy storage systems. Beyond energy storage, lithium compounds are used in pharmaceuticals for mood stabilization and in various industrial processes including glass and ceramic manufacturing, as well as aerospace alloys [1–6]. Globally, lithium deposits are unevenly distributed, with significant reserves located in the “Lithium Triangle” — comprising Chile, Argentina, and Bolivia — and Australia. Current estimates indicate that Chile holds the largest reserves, followed by Australia, Argentina, and China; the latter has risen to second place due to recent discoveries in the Qinghai-Tibet Plateau, with total global lithium deposits estimated to exceed 28 million metric tons (Mt) [7]. Lithium deposits are broadly categorized into three types: brine, pegmatite, and sedimentary deposits. Brine deposits, accounting for approximately 66% of global resources, occur in arid salt flats such as the Atacama Salar in Chile and are extracted via solar evaporation, benefiting from high lithium concentrations and low impurity levels. Pegmatite deposits, exemplified by the Greenbushes mine in Australia, contain lithium-rich minerals like spodumene and contribute about 26% of global production, despite higher extraction costs. Sedimentary deposits, including clay and evaporite formations such as La Ventana, are emerging as potential sources but remain underdeveloped owing to complex extraction challenges [4,8–13].

**Fig 1:**
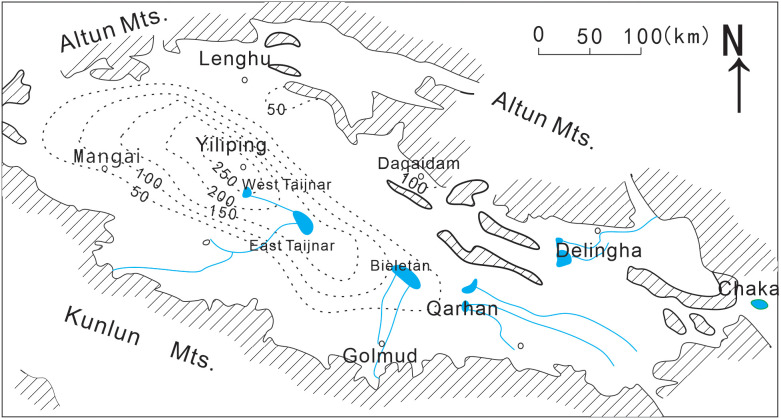
Map of the Qaidam basin, showing the variation in lithium content (ppm) in surface brines [14]. Blue lines are lakes and rivers; hashed areas are mountains.

The Qinghai-Tibet Plateau represents one of the world’s largest reserves of brine-type lithium resources, with the Qaidam Basin in Qinghai containing the most concentrated deposits. The lithium reserves (expressed as LiCl) are estimated to be approximately 15.207 million tons, primarily distributed in lithium-rich salt lakes such as Yiliping, East Taijinar, West Taijinar, and the Qarhan Salt Lake [14–16]. The spatial distribution of lithium within the Qaidam Basin salt lakes exhibits a distinct zonal pattern (Fig 1) [14]. The highest lithium concentrations are centered in the Yiliping Salt Lake, extending toward East Taijinar, West Taijinar, and the Bieletan section of Qarhan. Secondary lithium-enriched zones are identified near the Dachaidan Salt Lake, whereas the eastern salt lakes of the Qaidam Basin are characterized by relatively low lithium content. Since the 1980s, research on lithium in the brine systems of the Qaidam Basin has been progressively conducted. Existing studies indicate that the current understanding of lithium occurrence and behavior in this region remains incomplete. As lithium resources in salt lakes are predominantly found in surface and subsurface brines, previous investigations have mainly focused on dissolved lithium, leading to significant emphasis on extraction technologies and resource development [17–26]. In contrast, studies focusing on lithium within salt lake sediments have been limited. This gap has constrained the comprehension of the geochemical characteristics of lithium in sedimentary environments of the Qaidam Basin, which in turn is closely associated with the loss and inefficient utilization of lithium resources.

The earliest investigation of lithium in salt lake sediments of the Qaidam Basin was conducted by Zhang [14], whose analysis of soluble salts revealed higher lithium content in sulfate minerals compared to halite. However, the study did not provide detailed methodological descriptions regarding the dissolution process. In research on lithium isotope geochemistry in sediments from Dachaidan Salt Lake, Xiao et al. [27] determined lithium content using a 1:1 HCl solution as the solvent. Liu et al. [28] applied the Tessier sequential extraction method [29] to analyze lithium content across six different phases in sediments from the Qarhan Salt Lake, identifying the highest lithium concentration (17.80 ppm) in the residual phase, predominantly composed of clay minerals. Wang [30] reported lithium content in sediments of the Mahai Salt Lake, noting a maximum value of approximately 60 ppm with generally low levels overall, where the highest concentrations were mainly associated with clay and silt layers. Nevertheless, these previous studies have primarily focused on salt lakes with relatively low lithium content, leaving a notable research gap concerning lithium-rich (>80 ppm) areas within the Qaidam Basin, such as Yiliping, East Taijinar Salt Lake, West Taijinar Salt Lake, and the Bieletan region of Qarhan Salt Lake. To date, only Li [31] has examined water-soluble and acid-soluble (using 2% HNO₃) lithium in sediments from West Taijinar Salt Lake, suggesting that lithium in halite occurs mainly within inclusions. There remains a complete lack of related research on East Taijinar Lake, which hosts the highest lithium concentrations in the Qaidam Basin.

Furthermore, Li et al. [32] have emphasized the potential mineral resources present within salt lake sediments. A recent mineralogical investigation of three clay minerals in the Mahai Salt Lake (located in the Qaidam Basin) [33] revealed lithium concentrations that exceed both those in local solid salt layers and the regional geochemical background of bedrock, underscoring significant lithium enrichment within the clay deposits of the Qaidam Basin. To assess the resource potential, Pan et al. [34] completed 87 drill holes within a targeted area of the Mahai Salt Lake, with analytical results identifying a previously unrecognized clay-type sedimentary lithium deposit unique to the region [34]. A 2024 study further examined the mineral origins and mining conditions of the lithium-rich clay sequences in Mahai [35]. These findings collectively suggest that clay-type lithium deposits in salt lake environments represent a practically significant and novel form of lithium resource. However, these prior investigations have focused predominantly on salt lakes with low lithium concentrations and employed total lithium content measurements via sample digestion—a method impractical for real-world extraction due to its extensive solvent requirements. To address these research gaps, this study focuses on lithium-rich salt lakes within the Qaidam Basin to investigate the geochemical behavior of water-soluble lithium, with the aim of clarifying its distribution characteristics and mineralization potential in sedimentary contexts. Therefore, this study carries considerable academic and practical importance. Academically, it seeks to elucidate the geochemical features and occurrence mechanisms of water-soluble lithium in highly enriched saline lake sediments. Practically, the results are expected to provide essential insights for evaluating the mineralization potential and exploring sustainable resource replenishment pathways of clay-type lithium deposits in saline lacustrine systems, thereby bridging the gap between theoretical understanding and practical exploitation strategies.

## 2. Geology, materials and methods

### 2.1. Geological and geographic setting

The Qaidam Basin, located on the northeastern margin of the Qinghai–Tibet Plateau, constitutes a closed inland basin with an irregular rhomboidal shape. It is bounded by the Altun–Qilian Mountains to the north, the Ela Mountains to the east, and the Kunlun Mountains to the south [36,37]. Encompassing an area of approximately 126,000 km2, the basin exhibits elevations ranging from 2,600–3,200 meters, while the surrounding mountains exceed 4,000 meters, creating substantial topographic relief. This geomorphological setting, characterized by high mountains and deep basins, promotes rapid sediment accumulation, resulting in a thick succession of Meso-Cenozoic sedimentary deposits that reach up to 16,000 meters in thickness [38]. The salt lakes in the central and eastern parts of the basin, such as East Taijinar Salt Lake and Qarhan Salt Lake, are primarily recharged by rivers originating from the Kunlun Mountains. In contrast, the western region—characterized by a less developed river system sourced from the Altun Mountains—has experienced the early evaporation of numerous lakes, leading to the formation of saline-alkaline lands such as Kunteyi, Chahansilatu, and Dalangtan. Most lakes in the basin have evolved into salt lakes, with an average salinity of approximately 330 g/L [14].

**Fig 2:**
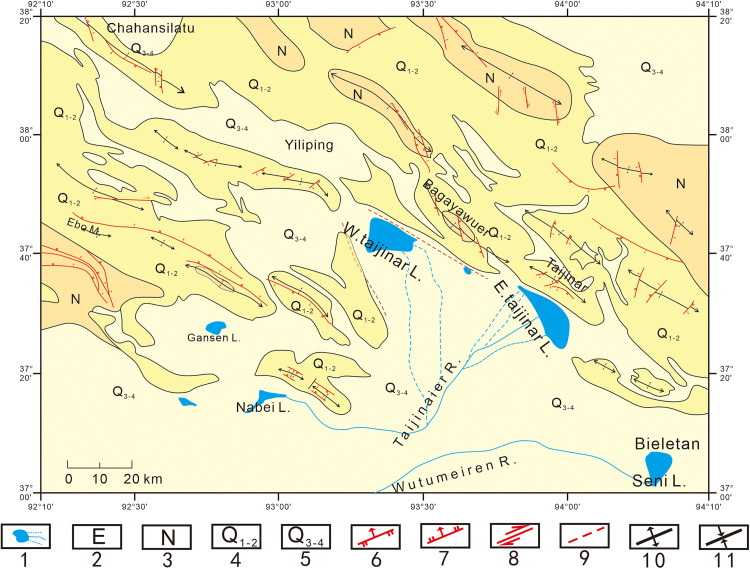
Map of major tectonic and sedimentologic features. Legend: 1. Lakes and rivers; 2. Neogene; 3. Palaeogene; 4. Early Pleistocene-Middle Pleistocene; 5. Epipleistocene–Holocene; 6. Reverse fault; 7. Normal fault; 8. Trike-slip fault; 9. Fault observed by remote sensing; 10. Anti-cline; and 11. Syncline [14,37–40].

In this study, the lithium-rich salt lakes in the Qaidam Basin comprise the Yiliping Salt Lake, East Taijinar Salt Lake, West Taijinar Salt Lake, and the Bieletan area of the Qarhan Salt Lake. This region is also identified as the depocenter of the Qaidam Basin [39]. Geologically, the lithium-rich salt lakes are situated within a Cenozoic faulted basin structure, characterized by anticlines and synclines formed in Paleogene and Quaternary systems (Fig 2). In terms of structural style, the fold axes in this area are predominantly oriented in a NW–SE direction. Faults are developed primarily along the margins of the basin and are mostly concealed, having been identified through seismic exploration [14,39].

### 2.2. Sample collection

**Fig 3:**
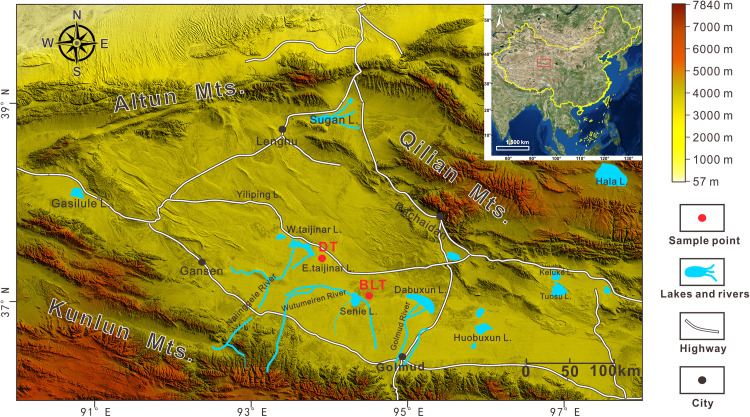
The location of sampling section in the Qaidam Basin (revised from Ma et al. [40]).

**Fig 4:**
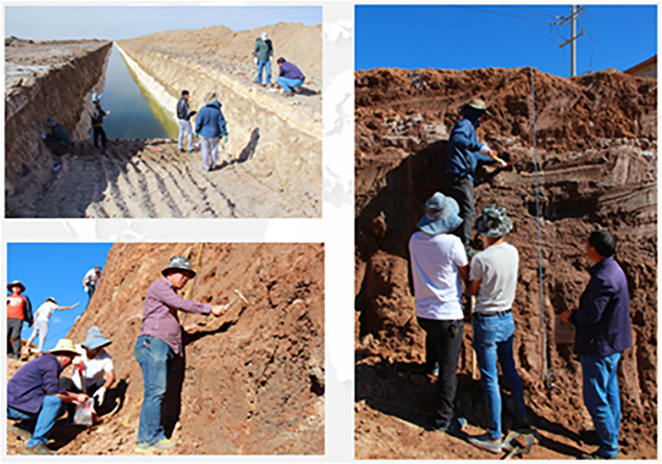
The photos in the field work.

During the 2017 field campaign targeting lithium-rich salt lakes in the Qaidam Basin, two representative sites were selected for sediment sampling: East Taijinar Salt Lake, which is renowned for containing the highest lithium concentrations in the basin and represents a typical lithium-rich brine system, and Bieletan, the only lithium-enriched zone within the Qarhan Salt Lake complex [14]. Together, these sites capture both the representative geochemical features and unique hydrological attributes of lithium-bearing salt lakes in the region. A total of 68 sediment samples were systematically collected from designated sections in East Taijinar Salt Lake and Bieletan (Fig 3). The sampling procedure involved immediately transferring sediments into chemically inert polyethylene bags to avoid cross-contamination. After collection, each bag was hermetically sealed with waterproof tape and labeled with essential metadata, including sampling coordinates, depth, sample number, and date (Fig 4 illustrates field conditions and methodologies) (The individual in this manuscript has given written informed consent (as outlined in PLOS consent form) to publish these photos). To maintain sample integrity and prevent microbial or chemical alteration, all samples were stored in a dry, low-humidity environment at temperatures below 4°C prior to analysis.

**Fig 5:**
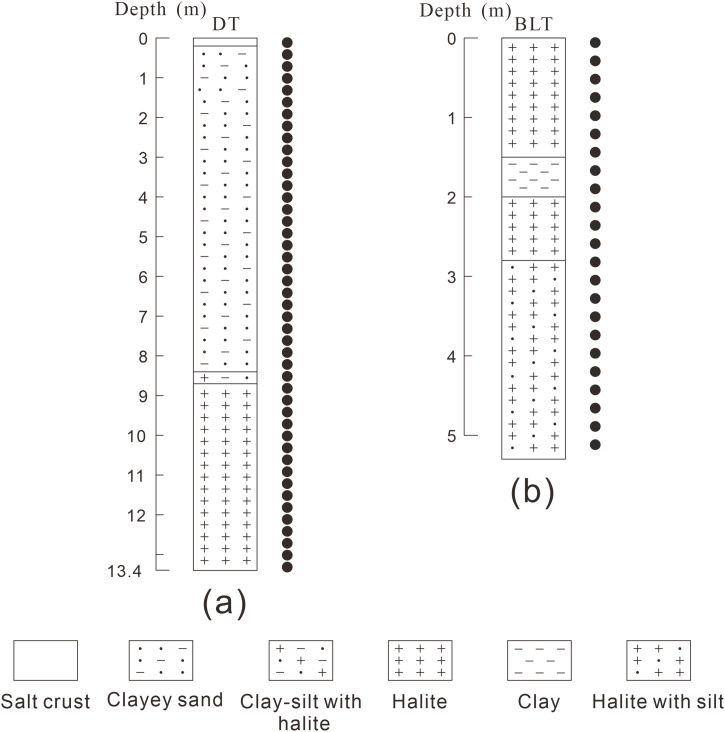
The sampling sites and lithology of different locations in the study area. Legend: a: the DT section, b: the BLT section; The black dots are samples.

In this study, two representative sections were investigated: the DT section, located within the East Taijinar Salt Lake, and the BLT section, situated in the Bieletan area. The geographical coordinates, altitude, and depth of each section are provided in Table 1. The lithological characteristics of each profile are summarized as follows (Fig 5):

**Table 1:** The location, elevation and depth of the sections in this study.

Section	Geographical coordinates	Altitude (m)	Depth (m)
DT	37°27’47.11“ N, 93°57’09.71” E	2686	13.4
BLT	37°03’57.75“ N, 94°34’33.29” E	2682	5.2

DT Section: Due to mineral resource development activities, a large portion of the East Taijinar Salt Lake became flooded by 2019, making it difficult to collect sediment samples from its sedimentary center. This constraint is one of the main reasons for the relatively limited research currently available on the East Taijinar Salt Lake. The samples from the DT section were collected in 2017. This section, which is an artificially excavated profile located in the sedimentary center of the lake [40], has a total depth of 13.4 m. Continuous, undisturbed salt lake sediment samples were retrieved from the section. The location and samples are highly representative, making research findings from this section reflective of the overall sedimentary characteristics of the East Taijinar Salt Lake. The lithology of the DT section, from top to bottom, is as follows: a salt crust from 0 to 0.2 m, with one sample collected at 0.1 m and an additional 45 XRD samples taken from 0.3 m to 13.4 m at intervals of 0.3 m; clayey sand from 0.2 to 8.4 m; clay-silt with white halite from 8.4 to 8.7 m; and white halite from 8.7 to 13.4 m (Fig 5a).

BLT Section: This section is situated in the Bieletan area, located in the western part of the Qarhan Salt Lake, with a total depth of approximately 5.2 m. A total of 23 sediment samples were collected from the profile beginning at a depth of 0.1 m and proceeding downward at intervals of 0.23 m. The samples were sequentially numbered from top to bottom as BLT01 to BLT23. The lithostratigraphy of the BLT section is divided into four distinct layers as follows: white halite from 0 to 1.5 m; clay from 1.5 to 2.0 m; white halite from 2.0 to 2.8 m; and white halite interbedded with silt from 2.8 to 5.2 m (Fig 5b).

### 2.3. Materials and methods

The filed word and analysis associated with this study was conducted under the full approval and oversight of the Qinghai Institute of Salt Lakes, Chinese Academy of Sciences. This permit ensures that the work adhered to the highest standards of academic ethics and integrity, and that the methodologies employed guarantee the reliability of the data and the reproducibility of the research. To ensure data reliability and representativeness, all analytical procedures for the collected samples were completed within three months after collection. This timeframe was strictly adhered to in order to minimize potential physicochemical alterations, such as changes in lithium speciation, moisture absorption, or microbial‐mediated transformations. Rapid processing helped preserve the original sedimentary properties, ensuring that the analytical outcomes accurately reflect in-situ conditions.

#### 2.3.1. X-ray diffraction (XRD).

To ensure the accuracy of XRD analysis, 45 samples from the DT section and 23 samples from the BLT section were first dried in an oven at approximately 60 °C. The dried samples were then ground into powder (<75 μm) using a ball mill. Subsequently, 3–5 grams of each powdered sample were weighed for further analysis. XRD measurements were performed at the Qinghai Institute of Salt Lakes, Chinese Academy of Sciences, using a PANalytical X’Pert PRO diffractometer (manufactured by PANalytical B.V., the Netherlands). The operating conditions included an X-ray tube voltage of 40 kV, a tube current of 35 mA, a scanning range of 3–70° (2θ), a step size of 0.016°, divergent (DS) and anti-scattering (SS) slits set at 1°, and a receiving slit (RS) aperture of 0.33 mm. The methodology employed in this study adheres to the standard titled “General rules for X-ray polycrystalline diffractometry”, with the standard number JY/T 0587–2020 [41].

#### 2.3.2. Element content analysis (ICP-OES).

A total of 68 sediment samples were collected from the DT and BLT sections. The samples were oven-dried and ground to a particle size of less than 75 μm. Approximately 1 g of each powdered sample was accurately weighed, mixed with 50 mL of distilled water, and subjected to continuous oscillation for 12 hours to ensure complete dissolution. After settling, the supernatant was carefully decanted for further chemical analysis. Elemental content analysis was conducted at the Qinghai Institute of Salt Lakes, Chinese Academy of Sciences. Lithium, boron, and potassium were quantified using an Inductively Coupled Plasma Optical Emission Spectrometer (ICP-OES, Avio 200, PerkinElmer, USA). The analytical method for quantitative determination of elements is based on “Analysis Methods for Brine and Salt, Third Edition” [42].

#### 2.3.3. Laser particle size analysis (LPSA).

Laser Scattering Particle Size Analysis (LSPA) is a technique developed in the 1970s and is now a standardized method under both ASTM (B822-20) and ISO (13322–1:2014) norms. In this study, a total of 28 samples were selected for LSPA: samples DT02 to DT27 from the clayey silt layer at depths between 0.2 and 8.2 m in the DT section, and samples BLT08 and BLT09 from the clay layer at 1.5–2.0 m depth in the BLT section. All analyses were conducted at the Qinghai Institute of Salt Lakes, Chinese Academy of Sciences, using a Malvern Mastersizer 2000 particle size analyzer (a UK-based company) with a measurement range of 0.02–2000 μm. The pretreatment and testing procedures were performed as follows: (a) Approximately 2.0 g of sample was weighed into a 50 mL beaker, treated with 10 mL of 10% H₂O₂ solution, and heated on a hotplate at 150 °C under continuous rinsing with ultrapure water until bubble formation ceased, ensuring complete removal of organic matter. (b) The temperature was reduced to 70 °C, and an adequate amount of 10% HCl solution was added under continuous stirring to dissolve carbonate components. (c) The sample was repeatedly rinsed with distilled water and decanted until the supernatant reached near neutrality. (d) After decantation, 10 mL of 0.05 mol/L (NaPO₃)₆ dispersant was added, and the mixture was subjected to ultrasonic vibration for 10 minutes to achieve full particle dispersion. (e) The particle size distribution of the ultrasonically treated suspension was measured. Parameters including obscuration, background signal, and residual error were monitored throughout the measurement. Samples showing abnormal values in these parameters were reprocessed and reanalyzed to ensure data accuracy. The analytical approach for the sample particle size was conducted by referring to the standard “Particle size analysis-Laser diffraction methods”. The standard number is ISO 13320:2020, IDT [43].

## 3. Discussion

### 3.1. Mineral composition and sedimentology

Salt lake sediments predominantly form in closed lake basins under arid to semi-arid climatic conditions. As lake water evolves from freshwater to saline water and eventually into brine, the associated sediments are primarily characterized by saline mineral deposition. However, natural salt lakes are not perfectly closed systems. Variations in tectonic activity and climate lead to fluctuations in water recharge and evaporation, resulting in modern salt lake sediments typically consisting of both chemical precipitates and clastic materials. Chemical deposits include minerals such as halite, mirabilite, gypsum, polyhalite, and bloedite, which precipitate directly from brine. Clastic deposits, transported by wind and fluvial processes into the basin, commonly contain quartz, albite, chlorite, muscovite, illite, and montmorillonite. This study examines two representative sections, DT and BLT, within the Qaidam Basin to analyze mineral composition and sedimentological characteristics. XRD results (Table 2) show that salt deposits in these lithium-rich lakes are dominated by halite and gypsum, with no polyhalite or carnallite detected. Notably, sylvite was identified for the first time in the East Taijinar Salt Lake, occurring from approximately 10.6 m to the deepest sampled depth of 13.4 m in the DT section. Carbonate minerals are primarily calcite and dolomite, with minor aragonite present in the upper part of the DT section. Clay minerals consist mainly of quartz, muscovite, albite, and chlorite, distributed extensively throughout both sections. The mineral assemblages in these two salt lakes are relatively simple, dominated by saline and clay minerals, with limited carbonate deposition.

**Table 2:** Identification results of minerals.

Section	Salt deposits	Clastic deposits
BLT	halite, gypsum, calcite, dolomite	quartz, albite, chlorite, muscovite
DT	halite, sylvine, gypsum, calcite, dolomite, aragonite	quartz, albite, chlorite, muscovite

#### 3.1.1. Mineral composition in the East Taijinar Salt Lake.

**Fig 6:**
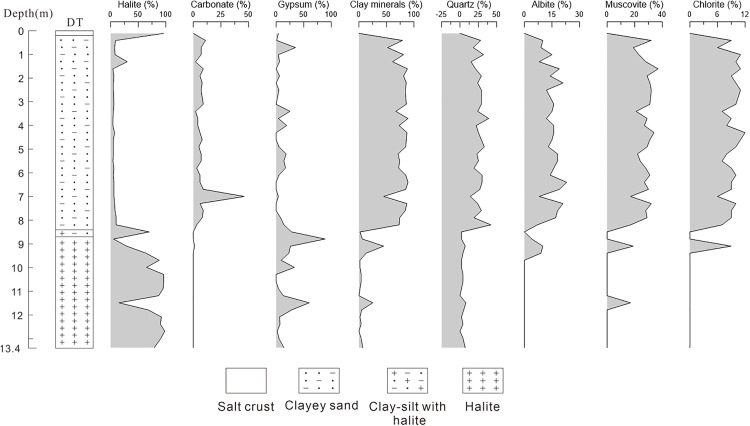
The comparison of mineral content in DT [40].

The DT section has a total depth of 13.4 m and is mineralogically composed mainly of halite, gypsum, and clay minerals, with minor amounts of carbonate minerals and sylvite (Fig 6 and S1 Table). In previous work [40], XRD analysis of the DT section was briefly reported in the context of reconstructing the formation and evolutionary history of the East Taijinar Salt Lake, focusing primarily on geochronological and sedimentological interpretations to infer developmental stages of the lake, with limited emphasis on detailed mineralogical characteristics, and without examining vertical zonation, compositional variability, or implications for lithium enrichment. The present study builds substantially on that foundation through a systematic mineralogical investigation. Based on distinct mineralogical characteristics, the section is divided into four stratigraphic layers: Layer 1 (0–0.2 m) consists of a salt crust; Layer 2 (0.2–8.5 m) is clayey silt; Layer 3 (8.5–8.7 m) comprises clay-silt interbedded with halite; and Layer 4 (8.7–13.4 m) is a dominant halite layer. The average halite content throughout the section is 32.31%, showing widespread distribution, although between 0.2 and 8.5 m, the content is significantly lower (5–7%). The average gypsum content is approximately 12.02%, peaking at 89% at 8.8 m depth. Carbonate minerals are primarily calcite and dolomite, with maximum contents of 7% and 43%, respectively, both occurring at 7.0 m depth. Aragonite is present only in the uppermost portion (0.2–0.7 m). Carbonate minerals occur predominantly within clastic layers and are scarce in halite-rich intervals. Clay minerals, averaging 6.85% in content, consist mainly of quartz, muscovite, albite, and clinochlore, and are primarily distributed within Layers 2 and 3. In Layer 1, halite constitutes 96% of the content, accompanied by 4% gypsum. Layer 2 is dominated by clay minerals (averaging 78%), with silicate minerals (quartz, albite, muscovite, chlorite) each ranging between 10% and 40%, along with lower halite, minor carbonates, and gypsum accounting for approximately 11%. Calcite remains below 10%, dolomite is generally below 5% except for a peak of 43% at 7.0 m, and aragonite is only detected in the top two samples at 7% and 4%. Layer 3 acts as a transitional zone with high salt content: one sample contains 70% halite and 28% gypsum with traces of quartz, while the other has 89% gypsum, 5% halite, and minor clay minerals. In Layer 4, halite predominates (average 72%, but decreasing noticeably at 11.5 m depth), gypsum averages 17%, carbonates are absent, and clay minerals occur in low concentrations. Notably, sylvite was identified between 10.6 m and 13.4 m (maximum is 15%), marking its first recorded occurrence in the East Taijinar Salt Lake. The observed sylvite-bearing layer has a minimum thickness of 2.8 m, and it is likely that its extent increases with depth, though deeper sampling was not possible due to the limited depth of the section.

#### 3.1.2. Mineral composition in the Bieletan.

**Fig 7:**
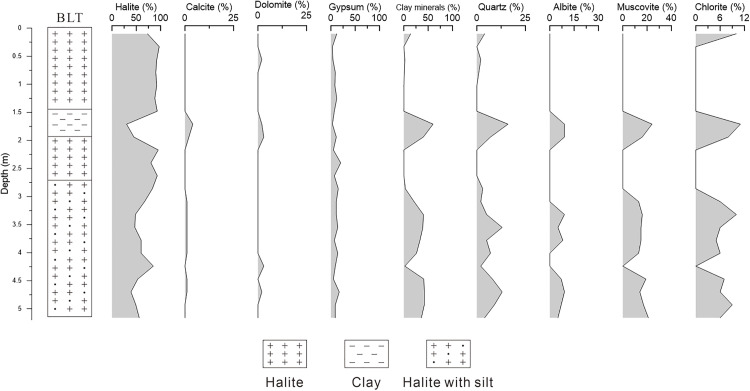
The comparison of mineral content in BLT.

Fig 7 and S2 Table show that the BLT section has a depth of 5.2 m, with a mineral composition dominated by halite, gypsum, and clay minerals, along with minor amounts of carbonate minerals. Based on distinct mineralogical characteristics, the section is divided into four layers: the first layer (0–1.5 m) consists of halite; the second layer (1.5–2.0 m) is composed of clay; the third layer (2.0–2.86 m) also comprises halite; and the fourth layer (2.86–5.2 m) consists of halite mixed with silt. The average gypsum content is approximately 9.65%, ranging from 7% to 12%, indicating relatively uniform distribution. Carbonate minerals are predominantly calcite, reaching a maximum content of 6%, and are notably less abundant in halite-rich layers, occurring mainly within the clay and silt intervals. Clay minerals have an average content of 19% and consist mainly of quartz, muscovite, albite, and clinochlore, distributed primarily within the 1.5–2.0 m and 2.86–5.2 m depth intervals. In the first layer, halite content is notably high, averaging over 89%, with gypsum as the second most abundant mineral at 7.8%. Carbonate minerals are scarce, with only one sample containing 2% dolomite, while clay minerals are also limited, reaching a maximum of 14% in one sample and remaining below 5% in others. In the second layer, halite content decreases significantly, falling below 50% in two samples, and clay minerals become predominant. Gypsum content remains similar to the upper layer, averaging around 10%, and carbonate minerals including calcite and dolomite appear, with total contents reaching 6% and 5%, respectively. The third layer is again dominated by halite, with an absence of clay and carbonate minerals. Gypsum content remains consistent with the second layer, showing no notable variation. The fourth layer is characterized as halite with silt, where halite constitutes more than 56% on average. Carbonate minerals such as calcite and dolomite are present in low amounts, none exceeding 3% per sample. Clay minerals represent the second most abundant component in this layer, averaging over 33% and consisting mainly of quartz and muscovite, followed by albite and chlorite. Gypsum content remains around 10%, consistent with the values observed in the overlying layers.

A comparison of the mineral compositions between the East Taijinar Salt Lake and the Bieletan area reveals that the East Taijinar Salt Lake exhibits greater mineralogical diversity. Notably, aragonite and sylvite are present in the East Taijinar Salt Lake but not identified in the Bieletan deposits. Of particular significance, this study provides the first documented report of sylvite deposition in the East Taijinar Salt Lake, with a minimum recorded thickness exceeding 2.8 meters.

### 3.2. Characteristic of water-soluble lithium in lithium-rich salt lake sediments

The enrichment of lithium in the salt lakes of the Qaidam Basin represents a distinctive feature that differentiates these systems from other salt lakes worldwide. While extensive research has been conducted on lithium in brine systems, significantly less attention has been given to lithium within associated salt lake sediments. Throughout the prolonged evolution of these hypersaline environments, lithium in the sediments has maintained a close genetic association with recharging water sources. Li [31] proposed that lithium enrichment in the brines of lakes recharged by the Nalinggele River—such as East Taijinar, West Taijinar, and Yiliping Salt Lakes—is driven by pulsed fluvial inputs under hyperarid climatic conditions, and that the accumulation of lithium in salt lake sediments occurs primarily through three mechanisms: adsorption onto clay minerals, entrapment within intercrystalline brines, and precipitation in evaporite minerals. To investigate the geochemical behavior of water-soluble lithium in sedimentary environments, this study utilizes aqueous extraction techniques on sediment samples collected from the East Taijinar Salt Lake and the Bieletan section of the Qarhan Salt Lake.

In salt lake sediments, water-soluble elements primarily originate from saline minerals such as halite and mirabilite, which readily dissolve in aqueous solutions. These elements constitute one major constituent phase of the readily extractable elements. Additionally, elements adsorbed onto mineral surfaces or within interlayers may be partially released into solution, although water-mediated desorption is generally limited to weakly bound fractions due to the mild extractive capacity of pure water. Although the two forms mentioned above are considered the dominant sources of water-soluble elements, the presence of slightly soluble minerals—such as gypsum—in salt lake sediments suggests that a minor portion of water-soluble elements may also be derived from the dissolution of such minimally soluble saline minerals.

#### 3.2.1. Distribution of water-soluble lithium in salt lake sediments.

**Fig 8:**
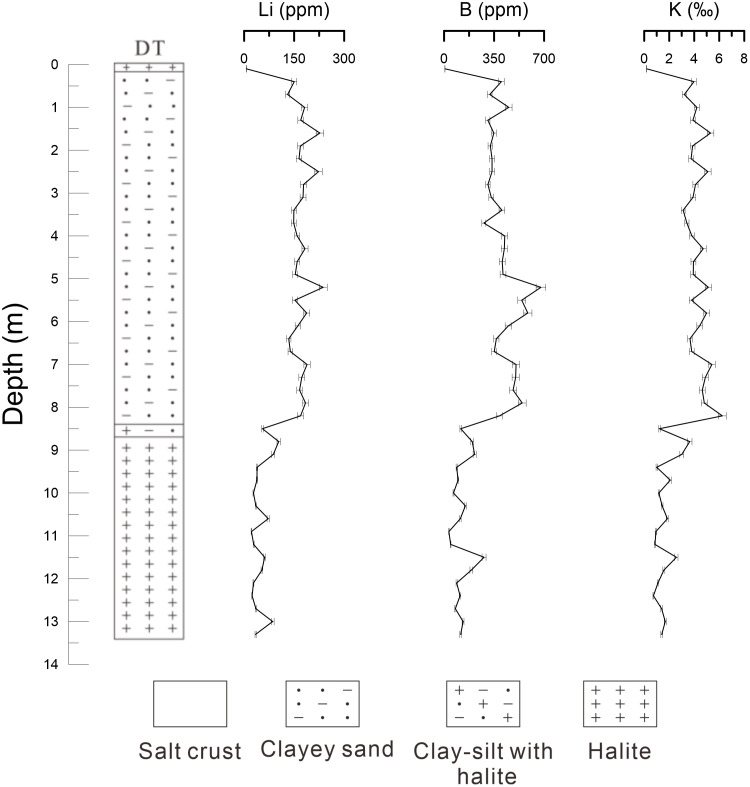
The comparison of lithium, boron and potassium content in DT.

As shown in S3 Table, the DT section in the East Taijinar Salt Lake exhibits average concentrations of 121 ppm for lithium, 296 ppm for boron, and 3.20‰ for potassium. Based on the vertical variation patterns of these three elements, the profile can be divided into three primary layers from bottom to top (Fig 8): 8.5–13.4 m, 0.2–8.5 m, and 0–0.2 m. The concentrations of lithium, boron, and potassium throughout the section display a consistent trend of initially increasing and subsequently decreasing upward. Lithologically, the uppermost layer (0–0.2 m), composed of salt crust, shows the lowest lithium content (6.37 ppm), with similarly minimal values for boron and potassium. The middle layer (0.2–8.5 m), consisting of clayey sand, contains the highest average lithium concentration (171 ppm), with pronounced enrichment intervals between 4.9–5.8 m and 6.7–8.2 m; the maximum lithium content within the entire section (recorded at 5.2 m) occurs within this clay-rich layer. The transitional interval from 8.5 to 8.7 m (clay-silt with white halite) has an average lithium content of 79.3 ppm. The basal layer (8.7–13.4 m), dominated by halite, averages 44.8 ppm lithium—significantly higher than the surface salt crust but markedly lower than the clayey middle layer.

These data reveal substantial lithium enrichment in clay-rich sand layers within the East Taijinar Salt, with negligible accumulation in halite-dominated intervals. Boron and potassium exhibit similar distribution patterns, suggesting comparable geochemical behavior among these three elements. All elements show their lowest concentrations in the surface salt crust (0–0.2 m), moderate enrichment in the deeper halite layer (8.7–13.4 m), and the highest values in the clay-rich middle layer (0.2–8.5 m). The strong coherence in their vertical distribution implies that water-soluble lithium, boron, and potassium in the sediments of East Taijinar Salt Lake likely share similar occurrence modes. This correlation further highlights the preferential association of these elements with clay-rich lithologies rather than highly soluble evaporites such as halite, likely governed by adsorption–desorption equilibria and mineralogical controls.

**Fig 9:**
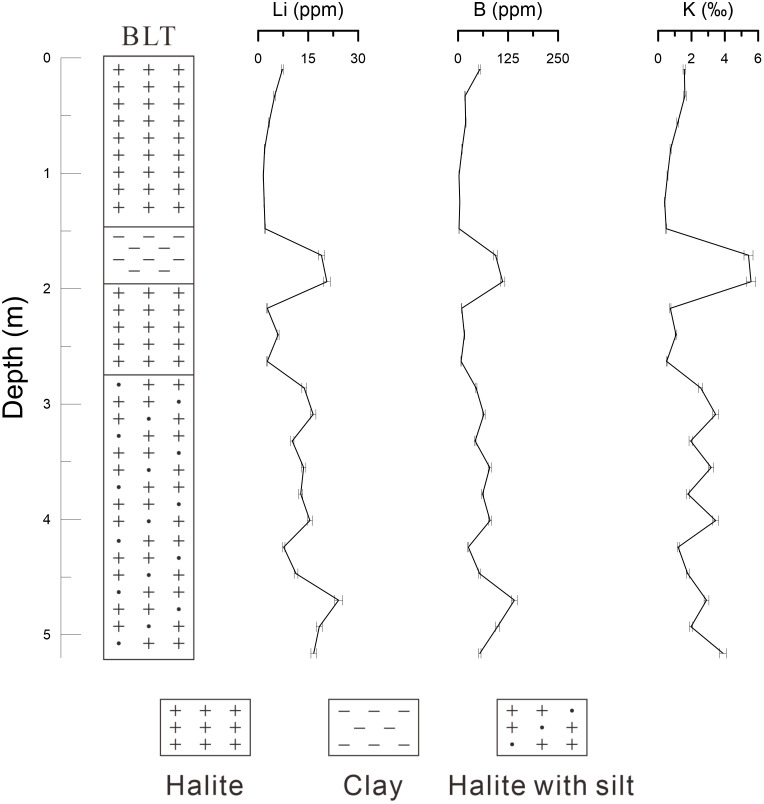
The comparison of lithium, boron and potassium content in BLT.

In the Bieletan area of the Qarhan Salt Lake, the measured average concentrations are 10.18 ppm for lithium, 47.50 ppm for boron, and 2.08‰ for potassium (S4 Table). Based on vertical variations in lithium content, the profile can be divided into four distinct layers (Fig 9): (1) 2.86–5.0 m, characterized by irregular fluctuations in lithium content; (2) 2.0–2.86 m, with consistently low lithium levels (<10 ppm); (3) 1.5–2.0 m, marked by significant lithium enrichment; and (4) 0–1.5 m, where lithium concentrations decrease to values comparable to those in layer (2). Both boron and potassium exhibit identical zonation and parallel trends, closely mirroring lithium’s behavior. Lithologically, the clay layer at 1.5–2.0 m contains the highest lithium concentration (19.8 ppm), while the halite-with-silt layer (2.86–5.0 m) shows variable lithium content averaging 14.7 ppm. Halite-dominated layers display minimal lithium enrichment: the upper halite layer (0–1.5 m) has the section’s lowest lithium concentration (3.25 ppm), and the halite layer from 2.0–2.86 m registers only slightly higher values (6.30 ppm). The distributions of boron and potassium align closely with lithium, peaking in the 1.5–2.0 m clay layer and reaching minima in the upper halite layer (0–1.5 m). These results demonstrate that water-soluble lithium, boron, and potassium in the Bieletan sediments preferentially concentrate in clay-rich lithologies, consistent with the distribution patterns observed in the East Taijinar Salt Lake, suggesting common enrichment mechanisms controlled by clay mineral interactions in evaporative environments.

In summary, water-soluble lithium in salt lake sediments is predominantly enriched in clay and clastic layers, with minimal accumulation in halite-dominated units. In the Qaidam Basin, the Bieletan and East Taijinar Salt Lake exhibit similar average potassium concentrations in their sediments (0.93‰ and 0.91‰, respectively). However, the Bieletan sediments show markedly lower average concentrations of both boron and lithium compared to those in the East Taijinar Salt Lake, with lithium content differing by more than an order of magnitude. This indicates significantly stronger lithium enrichment in the East Taijinar Salt Lake. Data from Li [31] further reveal intermediate water-soluble lithium levels in sediments from the West Taijinar Salt Lake (averaging ~78.4 ppm), which are higher than those in Bieletan but lower than in East Taijinar, suggesting a regional gradient in lithium enrichment among these interconnected saline systems.

**Fig 10:**
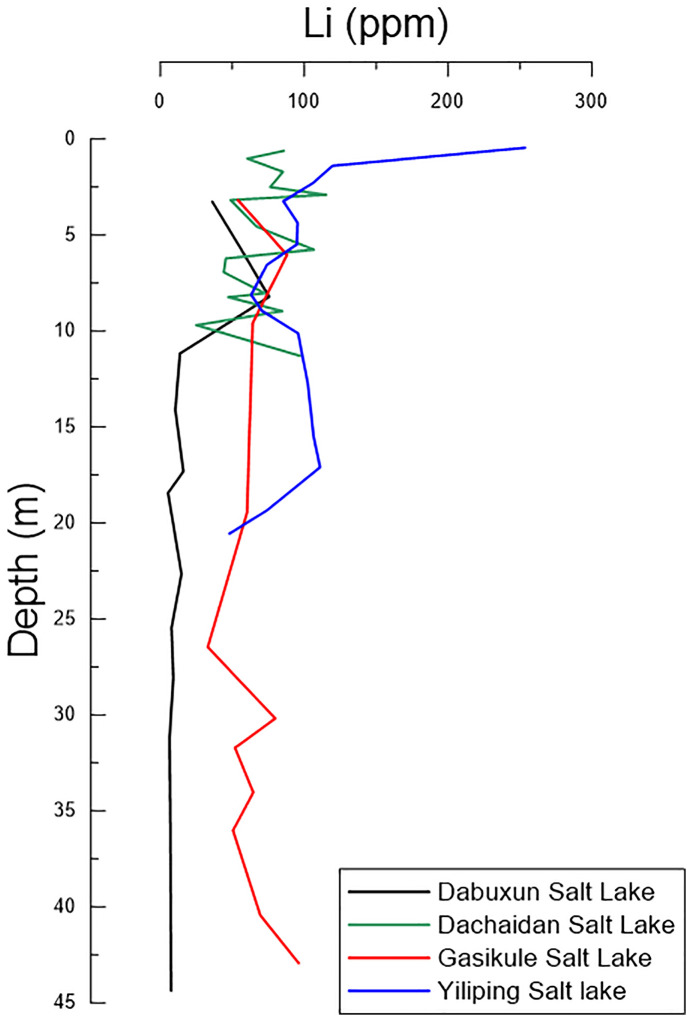
The comparison of Lithium content in Qarhan salt lake, Dachaidan salt lake, Gasikule salt lake and Yiliping salt lake [14].

According to previous studies on the distribution of water-soluble lithium in sediments from Dabuxun (Qarhan Salt Lake), Dachaidan Salt Lake, Gasikule Salt Lake, and Yiliping Salt Lake [14] (Fig 10), the Yiliping Salt Lake exhibits the highest concentrations, followed by Dachaidan Salt Lake and Gasikule Salt Lake, while Dabuxun shows the lowest values. However, specific quantitative data were not provided, making direct numerical comparisons of average concentrations impossible. Collectively, the spatial distribution of water-soluble lithium in the Qaidam Basin salt lake sediments correlates well with regional brine lithium enrichment patterns (Fig 1). Both records highlight a high-lithium zone centered around the Yiliping, West Taijinar, and East Taijinar Salt Lakes, with concentrations gradually decreasing outward from this core. This consistency underscores the dominant control of common hydrological and geochemical processes on lithium distribution across both brine and sedimentary reservoirs in arid evaporative settings.

#### 3.2.2. Occurrence forms of water-soluble lithium in salt lake sediments.

Three primary mechanisms have been proposed regarding the occurrence forms of water-soluble lithium in salt lake sediments: 1) Lithium encapsulated within fluid inclusions of saline minerals: During the crystallization of minerals such as halite (NaCl), lithium becomes trapped in microscopic fluid inclusions [14]; 2) Lithium adsorbed onto the surfaces of clay minerals: Lithium ions are retained primarily on the surfaces of muscovite, albite, and other clay minerals through adsorption [14]. Due to the limited capacity of water to desorb ions, this fraction consists mainly of weakly bound lithium; 3) Lithium entrained in porewater within sediments: Unconsolidated clastic sediments contain pore spaces filled with lithium-rich intercrystalline brine [31].

Li [31] inferred that lithium hosted in fluid inclusions within halite contributes minimally (7–85.6 ppm) to the total water-soluble lithium content in sediments of the West Taijinar Salt Lake, based on measurements from bulk halite samples. However, this estimation lacks direct evidence specifically quantifying lithium within fluid inclusions, underscoring the need for more precise analytical approaches. Several methods have been developed for accurate determination of lithium in halite fluid inclusions, including microdrilling-based ultra-microanalysis [44–45], laser ablation inductively coupled plasma mass spectrometry (LA-ICP-MS) [46–48], and SEM-EDS coupled with laser Raman spectroscopy [49–50]. Li et al. [51] applied LA-ICP-MS to analyze 12 halite samples from the ISL1A core in the Dabuxun area of the Qarhan Salt Lake and found highly variable lithium concentrations in fluid inclusions, ranging from 8.69 to 183.38 ppm. Their results indicated that upper halite layers (above 13 m depth) contained elevated lithium levels, with an average of 128.77 ppm across six samples, whereas deeper layers (13–31.52 m) exhibited significantly lower lithium values, averaging 14.57 ppm. Similarly, Hu et al. [52] used LA-ICP-MS to analyze the HC2105 core from the Yiliping Salt Lake and reported a lithium concentration of 17.78 ppm in one sample at 3.4 m depth, with six other samples exceeding 100 ppm. Although these studies indicate relatively high lithium concentrations in individual fluid inclusions, the overall contribution to bulk sediment lithium remains limited due to the sparse and heterogeneous distribution of such inclusions within halite matrices. Evaporation experiments conducted by Godfrey et al. [53] demonstrated minimal incorporation of lithium into halite during brine evaporation, a finding consistent with simulations based on seawater and Qinghai Lake brines [54–55]. In our study, halite-dominated layers in both the East Taijinar Salt Lake and the Bieletan area exhibit low water-soluble lithium concentrations—44.8 ppm and 3.25 ppm, respectively—which contrast sharply with clay-rich layers where lithium levels reach up to 171 ppm. Integrating previous research with our experimental data, it is concluded that lithium contained in fluid inclusions contributes only marginally to the total water-soluble lithium content in salt lake sediments.

Lithium present in brine entrapped within the pore spaces of unconsolidated sediments constitutes a component of water-soluble lithium. Modern brine in the East Taijinar Salt Lake contains lithium concentrations ranging from 150 to 344 ppm [14], while phreatic and confined intercrystalline brines in deeper layers exceed 600 ppm lithium [19], indicating high lithium concentrations not only in surface lake brines but also in subsurface brines that may infiltrate fine-grained clastic sediments. However, lithological analysis of the CK264 borehole by Ma et al. [40] indicates that the DT profile lacks phreatic brine, as surface brine overlies a 0.2–8.5 m thick aquiclude composed of clayey sand. The low permeability of this layer acts as an effective caprock for the confined brine reservoir, preventing significant infiltration of surface brine into the clayey sand. The lithium-containing entrapped brine in this context is represented by porewater within these clastic sediments, supported by measured moisture contents of 9–19% in this layer [40]. The 8.5–13.4 m halite layer corresponds to the confined brine lithium deposit, where intercrystalline brine in sediment pores contains 658–823 ppm lithium [19]. If pore brine lithium were the dominant source of water-soluble lithium, the halite layer (8.5–13.4 m) would be expected to exhibit higher lithium concentrations than the overlying clayey silt layer (0.2–8.5 m). Contrary to this expectation, the clayey silt layer shows the highest lithium enrichment (average 171 ppm), while the halite layer exhibits lower values (average 44.8 ppm). This discrepancy suggests that although lithium from pore brine contributes to the water-soluble fraction, it is not the primary form of occurrence. Instead, the elevated lithium content in clay-rich layers underscores the critical role of adsorption processes in controlling lithium distribution.

**Fig 11:**
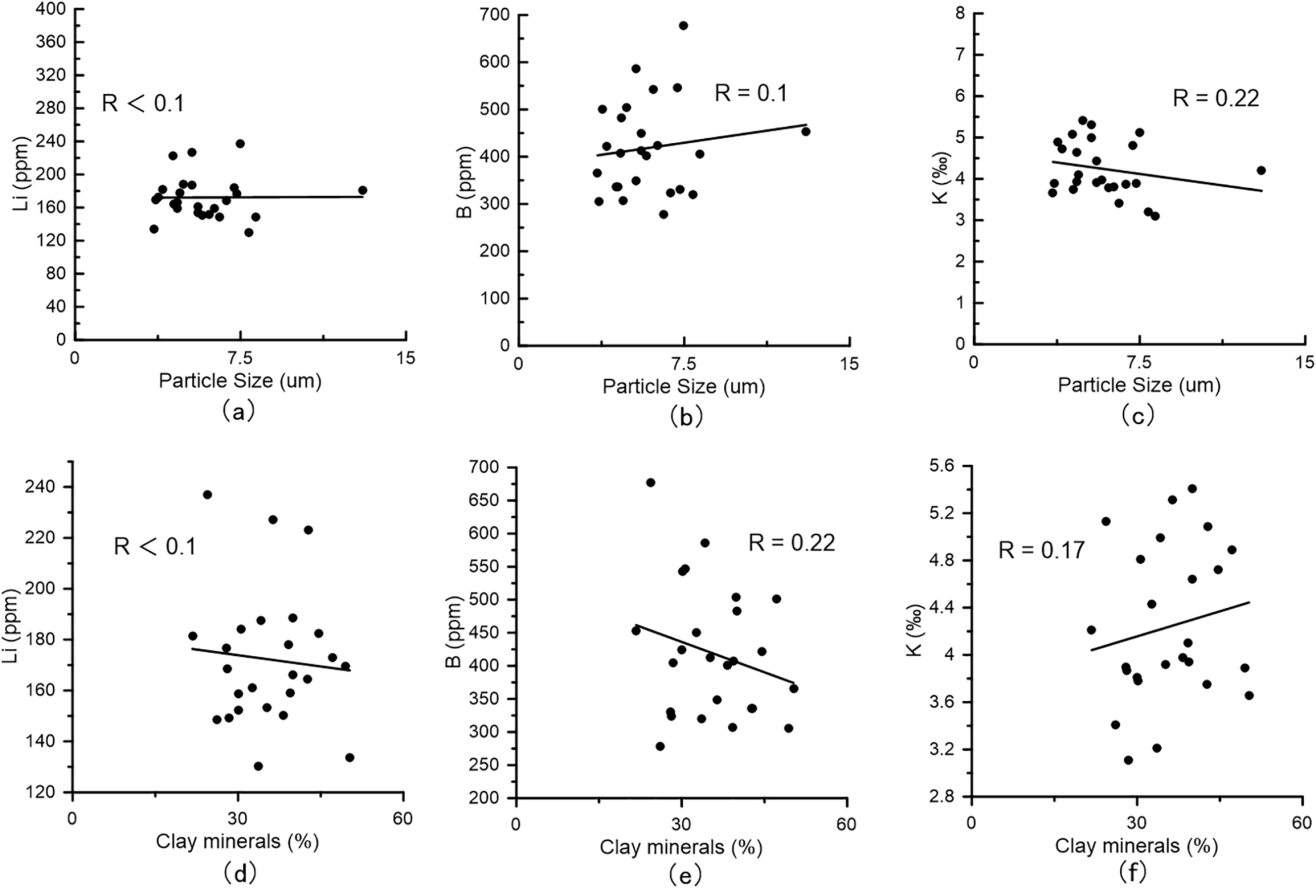
The linear correlation with lithium, boron, potassium and particle size, content of clay. a, b, c: The linear correlation with lithium, boron, potassium and particle size; d, e, f: The linear correlation with lithium, boron, potassium and content of clay (< 4 um).

Clay mineral-adsorbed lithium is acknowledged as a significant component of water-soluble lithium. Zhang [14] performed adsorption experiments which revealed that lithium adsorption on clay minerals increases with rising lithium concentrations in solution. Li [31] further clarified through evaporation experiments in the Nalinggele River that the lithium adsorption capacity of clay minerals is closely tied to their intrinsic properties once cation adsorption equilibrium is attained. In this study, the highest water-soluble lithium concentrations in both the East Taijinar Salt Lake and the Bieletan area consistently occur within clay-rich or clayey sand layers, underscoring a strong correlation between clay mineral content and water-soluble lithium enrichment. This supports the interpretation that lithium adsorbed onto clay minerals constitutes the predominant fraction of water-soluble lithium. However, due to the limited desorption capacity of water, only weakly adsorbed lithium is extracted during conventional water leaching, while strongly bound lithium remains insoluble. Previous studies have suggested that finer-grained sediments such as clay or silt exhibit enhanced adsorption capacities for trace elements [14,28]. To evaluate the influence of particle size on lithium adsorption in salt lake sediments, grain-size analysis was performed on 25 clayey sand samples from the DT profile in the East Taijinar Salt Lake. As shown in Fig 11, water-soluble lithium content displays only weak correlation with clay mineral particle size. Furthermore, linear regression analyses between the clay fraction (<4 μm) and lithium, boron, and potassium concentrations yield low correlation coefficients (R²). These results indicate that within the particle size range of the clayey sand layers in the East Taijinar Salt Lake, lithium adsorption—as well as that of boron and potassium—is not substantially influenced by grain size, but is likely governed by clay mineralogy and the ionic composition of the coexisting brine. This challenges the conventional view that finer particles inherently enhance adsorption capacity, highlighting instead the dominant role of mineral-specific surface interactions over purely physical textural controls in arid evaporitic environments.

**Fig 12:**
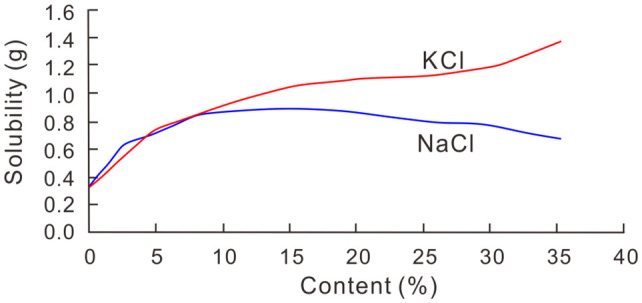
CaSO_4_ solubility at H_2_O and the effect of NaCl and KCl [56].

**Fig 13:**
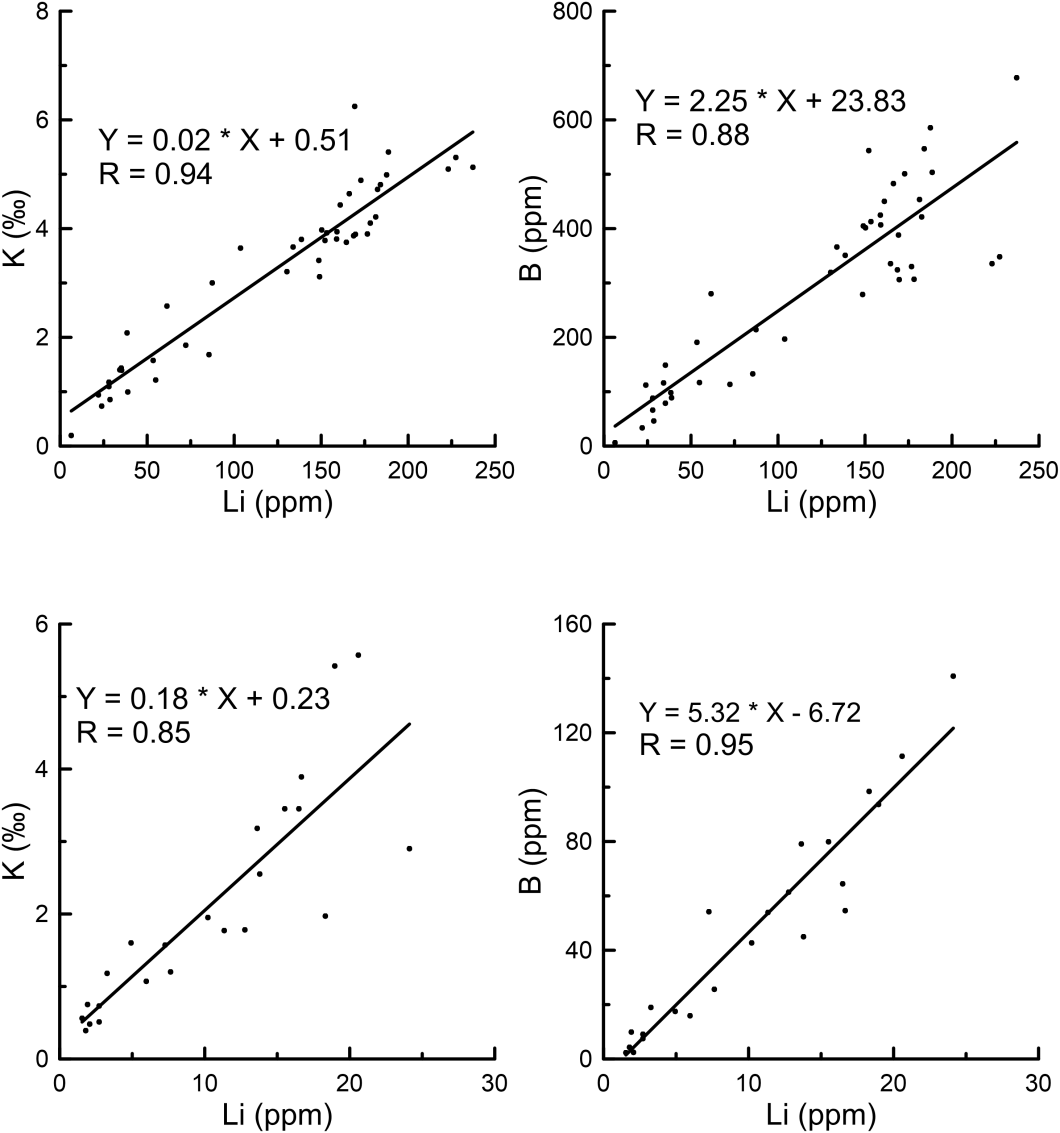
The linear correlation with lithium, boron and potassium. The upper is the linear correlation in DT; the lower is the linear correlation in BLT.

**Fig 14:**
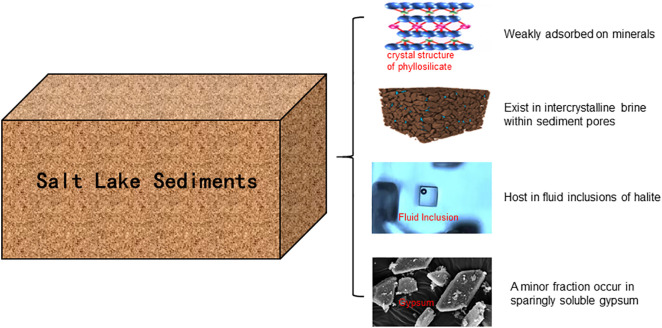
Occurrence Forms of Water-Soluble Lithium in Salt Lake Sediments.

In addition to the three previously recognized forms of water-soluble lithium in sediments, this study identifies a fourth occurrence: lithium associated with gypsum (CaSO₄·2H₂O). Although gypsum is traditionally classified as a sparingly soluble mineral, partial dissolution during water extraction can release structurally incorporated or adsorbed lithium. Experimental data from Yue et al. [56] indicate that calcium sulfate exhibits a solubility exceeding 2‰ at 20 °C, which further increases with rising NaCl concentrations (Fig 12). Given the hypersaline nature of the salt lake sediments examined in this study, the high NaCl content is likely to enhance gypsum solubility. Despite the limited solvent volumes used in standard dissolution protocols, the increased dissolution of gypsum under such saline conditions implies that lithium hosted within this mineral contributes a nonnegligible portion to the water-soluble fraction. Moreover, strong positive correlations (correlation coefficient R > 0.85) are observed among water-soluble lithium, potassium, and boron concentrations in both the East Taijinar Salt Lake and the Bieletan area (Fig 13), indicating shared geochemical behavior. These elements occur primarily as weakly adsorbed ions on clay mineral surfaces, with secondary contributions from pore brine electrolytes, lithium trapped in fluid inclusions of halite, and minor amounts released from partially dissolved gypsum. The coexistence of multiple occurrence forms—especially the previously underestimated contribution from gypsum-hosted lithium—highlights the complexity of water-soluble element speciation in salt lake sediments (Fig 14).

### 3.3. Resource potential for lithium in salt lake sediments

The rapid growth of the new energy sector has substantially increased global demand for lithium resources, making the assurance of a sustainable supply a critical challenge in recent years [7,9]. The finite nature of lithium reserves underscores the urgency to explore alternative sources. This study investigates the potential for lithium mineralization and replenishment in salt lake sediments by examining their lithium enrichment mechanisms and distribution patterns.

While the Qarhan Salt Lake represents a typical low-lithium brine system, lithium-rich salt lakes such as those in East Taijinar, West Taijinar, Yiliping, and the Bieletan area of Qarhan exhibit significantly greater potential for lithium resource sustainability. The lithium-enriched sediments in these regions, combined with active hydrological and diagenetic processes, may serve as dynamic buffers or even regenerative sources for brine lithium reservoirs. Lithium stored in clay-rich layers and intercrystalline brines within these sediments can be remobilized during brine recharge or evaporation cycles, effectively replenishing lithium in overlying or adjacent brine bodies. This mechanism highlights the dual role of sediments as both lithium sinks and geochemical reactors, presenting a strategic opportunity for sustainable lithium resource management in evaporitic basins.

Lithium resources in salt lakes are conventionally associated with surface or sub-surface brines, but lithium contained within sediments may represent a critical supplementary source for brine replenishment [33–35]. For instance, the Kunteyi Salt Lake, located in the western Qaidam Basin, is characterized by low lithium concentrations in its brines; however, its exceptionally thick sedimentary sequences are likely to host considerable lithium reserves. According to Li et al. [57], mineral exploration in this region has identified a halite reserve of 84.5 billion tons. Subsequent geochemical analysis of the ZK3208 borehole by Huang et al. [58] reported an average lithium content of 4.62 ppm within halite layers, equivalent to a total sedimentary lithium resource of approximately 390 kt. Similarly, Pan et al. [34] estimated total potential LiCl resources reaching 1.1441 million tons in the Mahai Salt Lake based on lithium concentrations measured through sediment digestion. The presence of such extensive salt lake sediments signifies residual resources beyond soluble mineral extraction, thereby extending the operational lifespan of the deposit [33–35,59]. While these salt lakes exemplify low-lithium brine systems, lithium-rich lakes such as those in East Taijinar, West Taijinar, Yiliping, and the Bieletan area of Qarhan demonstrate even greater potential. The lithium-enriched sediments in these areas, in conjunction with active hydrological and diagenetic processes, function as dynamic buffers or regenerative sources for brine lithium reservoirs. This mechanism underscores the dual role of sediments as both lithium sinks and potential suppliers, offering a strategic pathway toward sustainable lithium resource management in the Qaidam Basin.

The DT profile in the East Taijinar Salt Lake exhibits significant lithium enrichment within clayey sand layers, with an average water-soluble lithium concentration of 171 ppm, while the underlying clay-silt layers interbedded with halite contain 44.8 ppm lithium. Both values exceed the current lowest industrial grade for lithium (25 ppm). The feasibility of utilizing these sediments as a lithium resource also depends on the Mg/Li ratio, which averages 38.35 in water-soluble extracts (S5 Table)—a value comparable to that of actively mined brines in the same lake [17]. This similarity suggests that lithium from these sediments could serve as a supplementary resource during periods of brine lithium scarcity, especially given its substantial reserve volume. The use of water as a leaching solvent offers an economically viable and environmentally benign extraction method. Lithium-rich salt lake areas in the Qaidam Basin, such as those recharged by the Wutumiren River and Nalinggele River, possess abundant freshwater resources, resulting in low solvent acquisition costs and no risk of chemical contamination. Thus, water-soluble lithium in these enriched saline sediments holds considerable promise as a sustainable lithium resource. Additionally, the boron content in clay layers (415 ppm) significantly surpasses the cutoff grade (125 ppm), indicating potential for concurrent boron recovery. XRD analysis further identified a potassium-rich halite layer containing sylvite with a minimum thickness of 2.8 m, representing the first documented occurrence of sylvite deposition in the East Taijinar Salt Lake. These findings highlight the multi-resource potential of the lake’s sediments, with lithium, boron, and potassium collectively forming an exploitable resource assemblage.

In summary, lithium, boron, and potassium in the sediments of the East Taijinar Salt Lake demonstrate promising potential as recoverable resources within the salt lake system. During periods of reduced lithium availability in brines, the water extraction of these elements from sediments could offer a viable and supplementary approach to resource exploitation, supported by their considerable concentrations and favorable geochemical properties for straightforward and cost-effective recovery, thereby enhancing the sustainability of lithium production in the region.

**Perspectives:** This study provides preliminary insights into the occurrence mechanisms of water-soluble lithium in the lithium-rich salt lake sediments of the Qaidam Basin, pro-posing four hypothesized forms. However, these conclusions remain theoretical and require experimental validation. Future work will prioritize targeted mineral extraction techniques to confirm lithium distribution and refine its occurrence mechanisms. We plan to employ sequential dissolution methods to isolate lithium from specific mineral phases (e.g., halite, gypsum, and clay minerals), enabling quantitative analysis of its partitioning among different carriers. Advanced analytical tools, such as laser ablation inductively coupled plasma mass spectrometry (LA-ICP-MS) and synchrotron-based micro-X-ray fluorescence (μ-XRF), will further elucidate lithium’s micro-scale associations with host minerals. Additionally, long-term monitoring of lithium mobility in sediments under varying hydrological and climatic conditions could reveal dynamic interactions between brine recharge, evaporation cycles, and lithium retention. Environmental factors, such as pH and ionic strength, should also be investigated to assess their impact on lithium adsorption-desorption equilibria. Finally, integrating these findings with industrial practices—such as optimizing lithium recovery from low-grade sediments or co-extracting boron and potassium—will enhance the sustainable utilization of salt lake resources. By bridging theoretical models with practical validation, this research aims to establish a framework for lithium resource assessment and management in evaporitic basins, supporting global efforts toward green energy transitions.

**Perspectives:** This study provides preliminary insights into the occurrence mechanisms of water-soluble lithium in the lithium-rich salt lake sediments of the Qaidam Basin, proposing four hypothesized forms. However, these conclusions remain theoretical and require experimental validation. Future research will prioritize targeted extraction approaches to verify lithium distribution and clarify its specific host phases. We intend to apply sequential dissolution techniques to isolate lithium associated with distinct mineral carriers—such as halite, gypsum, and clay minerals—enabling quantitative assessment of its partitioning among these reservoirs. Advanced microanalytical methods, including laser ablation inductively coupled plasma mass spectrometry (LA-ICP-MS) and synchrotron-based micro-X-ray fluorescence (μ-XRF), will be employed to resolve lithium’s micro-scale spatial distribution and its associations with mineral substrates. Long-term monitoring of lithium mobility under varying hydrological and climatic conditions will further reveal dynamic processes linking brine recharge, evaporation cycles, and sediment-hosted lithium retention. The influence of environmental parameters—such as pH and ionic strength—on adsorption-desorption behavior should also be examined to better constrain lithium equilibria in these systems. Ultimately, integration of these insights with industrial practices—such as optimizing sediment-based lithium recovery or developing co-extraction strategies for boron and potassium—can promote sustainable resource utilization. By bridging theoretical models with empirical validation, this research aims to establish a comprehensive framework for lithium resource assessment and management in evaporitic basins, supporting global transition toward green energy.

## 4. Conclusions

The lithium-rich salt lake sediments of the Qaidam Basin exhibit distinct mineralogical and sedimentological characteristics, dominated by halite, gypsum, and clay minerals such as quartz, muscovite, and albite. A notable feature is the presence of sylvite (KCl) deposits exceeding 2.8 m in thickness in the East Taijinar Salt Lake—the first documented occurrence of such deposits in this region, underscoring its unique mineral diversity. Stratigraphically, the sediments are organized into clay-rich layers, exemplified by the 0.2–8.5 m interval in the East Taijinar Salt Lake, and halite-dominated layers, reflecting cyclical clastic input and evaporitic precipitation under arid conditions. Water-soluble lithium displays marked vertical and spatial zonation, with the highest concentrations occurring in clay layers and minimal enrichment in halite-rich intervals. Regionally, lithium enrichment exhibits a radiating pattern centered on high-concentration zones such as the Yiliping, East Taijinar, and West Taijinar Salt Lakes, consistent with the distribution of lithium in brines, and gradually decreases toward peripheral areas including the Bieletan section of the Qarhan Salt Lake.

The occurrence of water-soluble lithium in salt lake sediments is governed by four primary mechanisms: weak adsorption onto clay mineral surfaces, entrapment within intercrystalline pore brines, encapsulation in fluid inclusions of halite, and incorporation into the structure of gypsum. Among these, adsorption onto clay minerals dominates the occurrence, influenced more strongly by mineral-specific surface properties than by sediment particle size, as indicated by the low correlation between sediment particle size and lithium concentration. Although lithium is sporadically detected in fluid inclusions within halite, its overall contribution remains limited due to the sparse distribution of such inclusions. Lithium associated with gypsum, previously underestimated, becomes non-negligible under hypersaline conditions where gypsum solubility is enhanced. Strong correlations among lithium, boron, and potassium further highlight their shared geochemical behaviors and co-enrichment mechanisms in these sedimentary environments.

The resource potential of these sediments is substantial.. Lithium concentrations in clay-rich layers surpass the industrial cutoff grade, particularly in the East Taijinar Salt Lake, while the Mg/Li ratio (averaging 38.4) aligns with those of currently exploited brine resources, supporting feasible extraction during periods of brine lithium scarcity. Additionally, elevated boron content (415 ppm) and the presence of potassium-rich minerals such as sylvite further enhance the prospects for multi-element co-recovery. By utilizing these sediments as dynamic lithium reservoirs, the Qaidam Basin can significantly improve the sustainability and resilience of its lithium supply, offering a strategic resource base that aligns with global efforts toward clean energy transition.

## Supporting information

S1 Table Identification results in DT. (DOCX)

S2 Table Identification results in BLT. (DOCX)

S3 Table The content results of lithium, boron and potassium in DT. (DOCX)

S4 Table The content results of lithium, boron and potassium in BLT. (DOCX)

S5 Table The radio between Mg and Li in DT. (DOCX)
