# Supplementary material for: Characteristic and resource potential of water soluble lithium in lithium-rich salt lake sediments from Qaidam Basin, China
Source: PLoS One. 2025 Nov 7;20(11):e0336483. doi: 10.1371/journal.pone.0336483 (PMC12594433; doi:10.1371/journal.pone.0336483)
Supplement: S1 Table — (DOCX) [file pone.0336483.s001.docx]

**Table S1. Identification results in DT.**

| Sample ID | Depth (m) | Halite (%) | Sylvine (%) | Calcite (%) | Dolomite (%) | Aragonite (%) | Gypsum (%） | Quartz (%) | Albite (%) | Muscovite (%) | Chlorite (%) |
| --- | --- | --- | --- | --- | --- | --- | --- | --- | --- | --- | --- |
| DT01 | 0.1 | 96 |  |  |  |  | 4 |  |  |  |  |
| DT02 | 0.4 | 9 |  | 4 |  | 7 | 1 | 28 | 10 | 32 | 9 |
| DT03 | 0.7 | 7 |  | 3 |  | 4 | 35 | 18 | 9 | 19 | 6 |
| DT04 | 1 | 8 |  | 4 | 3 |  | 4 | 32 | 15 | 23 | 11 |
| DT05 | 1.3 | 30 |  | 2 |  |  | 6 | 15 | 8 | 28 | 10 |
| DT06 | 1.6 | 4 |  | 5 | 4 |  |  | 21 | 19 | 37 | 11 |
| DT07 | 1.9 | 6 |  | 4 | 2 |  | 6 | 29 | 14 | 29 | 9 |
| DT08 | 2.2 | 5 |  | 4 | 4 |  |  | 24 | 21 | 31 | 10 |
| DT09 | 2.5 | 5 |  | 5 | 2 |  | 5 | 29 | 12 | 32 | 11 |
| DT10 | 2.8 | 7 |  | 5 | 3 |  |  | 28 | 20 | 28 | 10 |
| DT11 | 3.1 | 6 |  | 6 | 3 |  |  | 29 | 16 | 31 | 10 |
| DT12 | 3.4 | 5 |  | 2 |  |  | 25 | 24 | 15 | 21 | 7 |
| DT13 | 3.7 | 4 |  | 2 | 2 |  | 4 | 39 | 12 | 29 | 9 |
| DT14 | 4 | 4 |  | 4 |  |  | 19 | 22 | 16 | 27 | 8 |
| DT15 | 4.3 | 7 |  | 6 |  |  |  | 25 | 16 | 34 | 12 |
| DT16 | 4.6 | 5 |  | 6 | 2 |  |  | 29 | 15 | 31 | 11 |
| DT17 | 4.9 | 5 |  | 5 |  |  | 5 | 33 | 13 | 29 | 10 |
| DT18 | 5.2 | 5 |  | 4 | 3 |  | 18 | 23 | 18 | 22 | 8 |
| DT19 | 5.5 | 4 |  | 7 |  |  | 14 | 25 | 18 | 24 | 7 |
| DT20 | 5.8 | 6 |  | 3 |  |  | 17 | 19 | 16 | 28 | 9 |
| DT21 | 6.1 | 5 |  | 6 |  |  | 5 | 30 | 14 | 31 | 10 |
| DT22 | 6.4 | 6 |  | 6 |  |  |  | 30 | 23 | 27 | 9 |
| DT23 | 6.7 | 6 |  | 7 | 2 |  |  | 27 | 19 | 30 | 9 |
| DT24 | 7 | 6 |  | 3 | 43 |  | 4 | 14 | 8 | 17 | 6 |
| DT25 | 7.3 | 7 |  | 6 |  |  |  | 24 | 21 | 32 | 10 |
| DT26 | 7.6 | 8 |  | 7 | 2 |  |  | 29 | 18 | 28 | 9 |
| DT27 | 7.9 | 10 |  | 5 | 3 |  | 8 | 19 | 17 | 29 | 9 |
| DT28 | 8.2 | 10 |  | 3 |  |  | 14 | 42 | 7 | 17 | 7 |
| DT29 | 8.5 | 70 |  |  |  |  | 28 | 2 |  |  |  |
| DT30 | 8.8 | 5 |  |  |  |  | 89 | 2 | 4 |  |  |
| DT31 | 9.1 | 29 |  | 1 |  |  | 26 | 7 | 10 | 19 | 9 |
| DT32 | 9.4 | 64 |  |  |  |  | 23 | 4 | 9 |  |  |
| DT33 | 9.7 | 88 |  |  |  |  | 9 | 2 |  |  |  |
| DT34 | 10 | 65 |  |  |  |  | 33 | 2 |  |  |  |
| DT35 | 10.3 | 96 |  |  |  |  |  | 4 |  |  |  |
| DT36 | 10.6 | 89 | 7 |  |  |  |  | 4 |  |  |  |
| DT37 | 10.9 | 85 | 10 |  |  |  | 4 | 2 |  |  |  |
| DT38 | 11.2 | 82 | 5 |  |  |  | 13 |  |  |  |  |
| DT39 | 11.5 | 15 |  |  |  |  | 60 | 8 |  | 17 |  |
| DT40 | 11.8 | 68 |  |  |  |  | 28 | 5 |  |  |  |
| DT41 | 12.1 | 86 | 6 |  |  |  | 6 | 2 |  |  |  |
| DT42 | 12.4 | 85 | 5 |  |  |  | 7 | 4 |  |  |  |
| DT43 | 12.7 | 91 | 7 |  |  |  | 2 |  |  |  |  |
| DT44 | 13 | 76 | 15 |  |  |  | 5 | 4 |  |  |  |
| DT45 | 13.4 | 74 | 5 |  |  |  | 14 | 7 |  |  |  |
