# Supplementary material for: Characteristic and resource potential of water soluble lithium in lithium-rich salt lake sediments from Qaidam Basin, China
Source: PLoS One. 2025 Nov 7;20(11):e0336483. doi: 10.1371/journal.pone.0336483 (PMC12594433; doi:10.1371/journal.pone.0336483)
Supplement: S2 Table — (DOCX) [file pone.0336483.s002.docx]

**Table S2. Identification results in BLT.**

| Sample ID | Depth (m) | Halite (%) | Calcite (%) | Dolomite (%) | Gypsum (%） | Quartz (%） | Albite (%) | Muscovite (%) | Chlorite (%) |
| --- | --- | --- | --- | --- | --- | --- | --- | --- | --- |
| BLT01 | 0.1 | 73 |  |  | 12 | 4 |  |  | 10 |
| BLT02 | 0.33 | 97 |  |  | 3 |  |  |  |  |
| BLT03 | 0.56 | 92 |  | 2 | 4 | 2 |  |  |  |
| BLT04 | 0.79 | 90 |  |  | 9 | 1 |  |  |  |
| BLT05 | 1.02 | 92 |  |  | 8 |  |  |  |  |
| BLT06 | 1.25 | 88 |  |  | 12 |  |  |  |  |
| BLT07 | 1.48 | 93 |  |  | 7 |  |  |  |  |
| BLT08 | 1.71 | 30 | 4 | 2 | 3 | 16 | 9 | 24 | 11 |
| BLT09 | 1.94 | 45 | 2 | 3 | 11 | 7 | 9 | 16 | 8 |
| BLT10 | 2.17 | 95 |  |  | 5 |  |  |  |  |
| BLT11 | 2.4 | 80 |  |  | 20 |  |  |  |  |
| BLT12 | 2.63 | 93 |  |  | 7 |  |  |  |  |
| BLT13 | 2.86 | 83 |  |  | 15 | 3 |  |  |  |
| BLT14 | 3.09 | 67 | 1 |  | 11 | 2 |  | 13 | 6 |
| BLT15 | 3.32 | 48 | 1 |  | 11 | 5 | 9 | 16 | 10 |
| BLT16 | 3.55 | 47 | 1 |  | 14 | 13 | 5 | 15 | 6 |
| BLT17 | 3.78 | 60 | 1 |  | 7 | 5 | 8 | 15 | 5 |
| BLT18 | 4.01 | 60 | 1 |  | 14 | 7 |  | 13 | 6 |
| BLT19 | 4.24 | 85 |  | 3 | 9 | 2 |  |  |  |
| BLT20 | 4.47 | 53 | 1 |  | 5 | 8 | 7 | 19 | 7 |
| BLT21 | 4.7 | 39 | 1 | 2 | 17 | 13 | 9 | 14 | 6 |
| BLT22 | 4.93 | 49 |  |  | 9 | 9 | 7 | 17 | 9 |
| BLT23 | 5.16 | 56 |  |  | 9 | 4 | 5 | 21 | 6 |
