# Supplementary material for: Characteristic and resource potential of water soluble lithium in lithium-rich salt lake sediments from Qaidam Basin, China
Source: PLoS One. 2025 Nov 7;20(11):e0336483. doi: 10.1371/journal.pone.0336483 (PMC12594433; doi:10.1371/journal.pone.0336483)
Supplement: S3 Table — (DOCX) [file pone.0336483.s003.docx]

**Table S3. The content results of lithium, boron and potassium in DT.**

| Sample ID | Depth (m) | Li (ppm) | B (ppm) | K (‰) | Sample ID | Depth (m) | Li (ppm) | B (ppm) | K (‰) |
| --- | --- | --- | --- | --- | --- | --- | --- | --- | --- |
| DT01 | 0.1 | 6.37 | 5.82 | 0.19 | DT24 | 7.0 | 189 | 504 | 5.41 |
| DT02 | 0.4 | 150 | 401 | 3.98 | DT25 | 7.3 | 173 | 501 | 4.89 |
| DT03 | 0.7 | 130 | 320 | 3.21 | DT26 | 7.6 | 166 | 483 | 4.64 |
| DT04 | 1.0 | 181 | 453 | 4.21 | DT27 | 7.9 | 184 | 547 | 4.81 |
| DT05 | 1.3 | 170 | 306 | 3.89 | DT28 | 8.2 | 169 | 388 | 6.25 |
| DT06 | 1.6 | 227 | 348 | 5.31 | DT29 | 8.5 | 54.9 | 117 | 1.21 |
| DT07 | 1.9 | 169 | 324 | 3.87 | DT30 | 8.7 | 104 | 197 | 3.64 |
| DT08 | 2.2 | 165 | 335 | 3.75 | DT31 | 9.1 | 87.4 | 214 | 3.00 |
| DT09 | 2.5 | 223 | 336 | 5.09 | DT32 | 9.4 | 38.8 | 88.6 | 1.00 |
| DT10 | 2.8 | 178 | 307 | 4.10 | DT33 | 9.7 | 38.4 | 97.9 | 2.08 |
| DT11 | 3.1 | 177 | 330 | 3.90 | DT34 | 10.0 | 28.0 | 65.8 | 1.17 |
| DT12 | 3.4 | 149 | 405 | 3.11 | DT35 | 10.3 | 35.2 | 149 | 1.43 |
| DT13 | 3.7 | 149 | 279 | 3.41 | DT36 | 10.6 | 72.3 | 114 | 1.85 |
| DT14 | 4.0 | 159 | 425 | 3.81 | DT37 | 10.9 | 22.0 | 33.7 | 0.94 |
| DT15 | 4.3 | 183 | 422 | 4.72 | DT38 | 11.2 | 28.6 | 46.0 | 0.86 |
| DT16 | 4.6 | 159 | 407 | 3.94 | DT39 | 11.5 | 61.5 | 280 | 2.58 |
| DT17 | 4.9 | 153 | 413 | 3.92 | DT40 | 11.8 | 53.3 | 191 | 1.57 |
| DT18 | 5.2 | 237 | 678 | 5.13 | DT41 | 12.1 | 28.0 | 88.2 | 1.10 |
| DT19 | 5.5 | 152 | 543 | 3.78 | DT42 | 12.4 | 24.0 | 112 | 0.74 |
| DT20 | 5.8 | 188 | 586 | 4.99 | DT43 | 12.7 | 35.2 | 78.7 | 1.39 |
| DT21 | 6.1 | 161 | 450 | 4.43 | DT44 | 13 | 85.6 | 133 | 1.68 |
| DT22 | 6.4 | 134 | 366 | 3.66 | DT45 | 13.4 | 34.3 | 116 | 1.40 |
| DT23 | 6.7 | 139 | 351 | 3.80 | Average |  | 121 | 296 | 3.20 |
