# Supplementary material for: Characteristic and resource potential of water soluble lithium in lithium-rich salt lake sediments from Qaidam Basin, China
Source: PLoS One. 2025 Nov 7;20(11):e0336483. doi: 10.1371/journal.pone.0336483 (PMC12594433; doi:10.1371/journal.pone.0336483)
Supplement: S4 Table — (DOCX) [file pone.0336483.s004.docx]

**Table S4. The content results of lithium, boron and potassium in BLT.**

| Sample ID | Depth (m) | Li (ppm) | B (ppm) | K (‰) | Sample ID | Depth (m) | Li (ppm) | B (ppm) | K (‰) |
| --- | --- | --- | --- | --- | --- | --- | --- | --- | --- |
| BLT01 | 0.1 | 7.26 | 54.2 | 1.57 | BLT13 | 2.86 | 13.8 | 45.0 | 2.55 |
| BLT02 | 0.33 | 4.93 | 17.5 | 1.60 | BLT14 | 3.09 | 16.5 | 64.4 | 3.45 |
| BLT03 | 0.56 | 3.27 | 19.0 | 1.18 | BLT15 | 3.32 | 10.2 | 42.7 | 1.95 |
| BLT04 | 0.79 | 1.92 | 9.90 | 0.75 | BLT16 | 3.55 | 13.6 | 79.1 | 3.18 |
| BLT05 | 1.02 | 1.54 | 2.28 | 0.56 | BLT17 | 3.78 | 12.8 | 61.3 | 1.78 |
| BLT06 | 1.25 | 1.79 | 4.24 | 0.39 | BLT18 | 4.01 | 15.5 | 79.9 | 3.45 |
| BLT07 | 1.48 | 2.06 | 2.40 | 0.48 | BLT19 | 4.24 | 7.65 | 25.6 | 1.20 |
| BLT08 | 1.71 | 19.0 | 93.7 | 5.42 | BLT20 | 4.47 | 11.4 | 53.9 | 1.77 |
| BLT09 | 1.94 | 20.6 | 111 | 5.57 | BLT21 | 4.70 | 24.1 | 141 | 2.90 |
| BLT10 | 2.17 | 2.71 | 9.06 | 0.73 | BLT22 | 4.93 | 18.3 | 98.4 | 1.97 |
| BLT11 | 2.40 | 5.97 | 15.8 | 1.07 | BLT23 | 5.16 | 16.7 | 54.5 | 3.89 |
| BLT12 | 2.63 | 2.72 | 7.51 | 0.51 | Average |  | 10.2 | 47.5 | 2.08 |
