# Supplementary material for: Characteristic and resource potential of water soluble lithium in lithium-rich salt lake sediments from Qaidam Basin, China
Source: PLoS One. 2025 Nov 7;20(11):e0336483. doi: 10.1371/journal.pone.0336483 (PMC12594433; doi:10.1371/journal.pone.0336483)
Supplement: S5 Table — (DOCX) [file pone.0336483.s005.docx]

**Table S5. The radio between Mg and Li in DT.**

| Sample ID | Depth (m) | Magnesium (%) | Li (ppm) | Mg/Li | Sample ID | Depth (m) | Magnesium (%) | Li (ppm) | Mg/Li |
| --- | --- | --- | --- | --- | --- | --- | --- | --- | --- |
| DT01 | 0.1 | 0.06 | 6.37 | 94.2 | DT24 | 7 | 0.45 | 189 | 23.9 |
| DT02 | 0.4 | 0.35 | 150 | 23.3 | DT25 | 7.3 | 0.39 | 173 | 22.6 |
| DT03 | 0.7 | 0.32 | 130 | 24.6 | DT26 | 7.6 | 0.43 | 166 | 25.9 |
| DT04 | 1 | 0.49 | 181 | 27.0 | DT27 | 7.9 | 0.42 | 184 | 22.8 |
| DT05 | 1.3 | 0.52 | 170 | 30.7 | DT28 | 8.2 | 0.42 | 169 | 24.8 |
| DT06 | 1.6 | 0.64 | 227 | 28.2 | DT29 | 8.5 | 0.19 | 54.9 | 34.6 |
| DT07 | 1.9 | 0.48 | 169 | 28.5 | DT30 | 8.8 | 0.39 | 104 | 37.6 |
| DT08 | 2.2 | 0.41 | 165 | 24.9 | DT31 | 9.1 | 0.33 | 87.4 | 37.8 |
| DT09 | 2.5 | 0.57 | 223 | 25.6 | DT32 | 9.4 | 0.16 | 38.8 | 41.2 |
| DT10 | 2.8 | 0.45 | 178 | 25.3 | DT33 | 9.7 | 0.19 | 38.4 | 49.5 |
| DT11 | 3.1 | 0.45 | 177 | 25.5 | DT34 | 10 | 0.19 | 28.0 | 67.9 |
| DT12 | 3.4 | 0.4 | 149 | 26.8 | DT35 | 10.3 | 0.17 | 35.2 | 48.3 |
| DT13 | 3.7 | 0.4 | 149 | 26.9 | DT36 | 10.6 | 0.25 | 72.3 | 34.6 |
| DT14 | 4 | 0.43 | 159 | 27.1 | DT37 | 10.9 | 0.12 | 22.0 | 54.6 |
| DT15 | 4.3 | 0.42 | 183 | 23.0 | DT38 | 11.2 | 0.47 | 28.6 | 165 |
| DT16 | 4.6 | 0.34 | 159 | 21.4 | DT39 | 11.5 | 0.24 | 61.5 | 39.1 |
| DT17 | 4.9 | 0.38 | 153 | 24.8 | DT40 | 11.8 | 0.22 | 53.3 | 41.2 |
| DT18 | 5.2 | 0.58 | 237 | 24.5 | DT41 | 12.1 | 0.18 | 28.0 | 64.3 |
| DT19 | 5.5 | 0.39 | 152 | 25.6 | DT42 | 12.4 | 0.15 | 24.0 | 62.6 |
| DT20 | 5.8 | 0.51 | 188 | 27.2 | DT43 | 12.7 | 0.27 | 35.2 | 76.6 |
| DT21 | 6.1 | 0.39 | 161 | 24.2 | DT44 | 13 | 0.35 | 85.6 | 40.9 |
| DT22 | 6.4 | 0.35 | 134 | 26.2 | DT45 | 13.4 | 0.17 | 34.3 | 49.5 |
| DT23 | 6.7 | 0.36 | 139 | 26.0 | Average |  | 0.35 | 121 | 38.4 |
